# HSP27 Attenuates cGAS-Mediated IFN-β Signaling through Ubiquitination of cGAS and Promotes PRV Infection

**DOI:** 10.3390/v14091851

**Published:** 2022-08-23

**Authors:** Xiangrong Li, Jingying Xie, Dianyu Li, Hongshan Li, Yuhui Niu, Bei Wu, Yanmei Yang, Zhenfang Yan, Xiangbo Zhang, Lei Chen, Ruofei Feng

**Affiliations:** 1 Key Laboratory of Biotechnology and Bioengineering of State Ethnic Affairs Commission, Biomedical Research Center, Northwest Minzu University, Lanzhou 730030, China; 2 Gansu Tech Innovation Center of Animal Cell, Biomedical Research Center, Northwest Minzu University, Lanzhou 730030, China; 3 College of Life Science and Engineering, Northwest Minzu University, Lanzhou 730030, China

**Keywords:** pseudorabies virus, heat shock protein 27, cyclic GMP-AMP synthase, cGAS-STING signaling pathway, ubiquitination

## Abstract

Pseudorabies (PR) is a domestic and wild animal infectious disease caused by the pseudorabies virus (PRV) and is one of the major infectious diseases that endanger the global swine industry. Studies have reported that PRV may achieve cross-species transmission from pigs to humans in recent years. Therefore, in-depth exploration of the relationship between PRV and host proteins is of great significance for elucidating the pathogenic mechanism of PRV and anti-PRV infection. Here, we report that heat shock protein 27 (HSP27) ubiquitinates and degrades cyclic GMP-AMP synthase (cGAS) and attenuates cGAS-mediated antiviral responses, thereby promoting PRV infection. Overexpression of HSP27 promoted PRV proliferation in vitro, while knockdown of HSP27 inhibited PRV infection. Importantly, we found that HSP27 inhibited PRV infection or poly(dA:dT)-activated IFN-β expression. Further studies found that HSP27 may inhibit cGAS-STING-mediated IFN-β expression through targeting cGAS. In addition, we found that HSP27 can suppress the expression of endogenous cGAS in different cells at both gene transcription and protein expression levels, and that HSP27 interacts with and ubiquitinates cGAS. In conclusion, we reveal for the first time that HSP27 is a novel negative regulator of the cGAS-STING signaling pathway induced by PRV infection or poly(dA:dT) activation and demonstrate that HSP27 plays a crucial role in PRV infection.

## 1. Introduction

Innate immune response is the body’s first line of defense against viral infection. Virus infection could induce host innate immune responses, and this plays an important as well as decisive role in the outcome of the infected host. The crucial point is that the host can establish an antiviral status to antagonize the invasion of the virus via identifying the components of invading virus through pattern recognition receptors (PRRs), which activate signaling pathways to produce type I interferons (IFN-I) [1]. The released IFN-I then binds to IFN receptors (IFNAR1 and/or IFNAR2) to activate the downstream JAK-STAT signaling pathway, eventually leading to the expression of a series of interferon-stimulated genes (ISGs). ISGs could realize numerous cellular consequences, including antiviral defense, antiproliferative activities, and stimulation of adaptive immunity [2,3,4].

For DNA virus, Toll-like receptor 9 (TLR9), cyclic GMP-AMP (cGAMP) synthase (cGAS), DAI (DLM-1/ZBP1), absent in melanoma 2 (AIM2), and IFN gamma-inducible protein 16 (IFI16) serve as the main PRRs that recognize viral DNA [5,6]. PRRs then recruit a series of signal transduction molecules, such as myeloid differentiation primary response gene 88 (MyD88), mitochondrial antiviral-signaling protein (MAVS), intracellular stimulator of IFN genes (STING). These proteins then transfer the different signals to the downstream molecules in different signaling pathways, which eventually lead to activation and translocation of several transcription factors, including NF-κB, interferon regulatory factor 3 (IRF3), and IRF7 into the nucleus to induce the expression of IFN-I and proinflammatory cytokines [7,8,9].

Pseudorabies virus (PRV) is a typical DNA virus. It belongs to the family *Herpesviridae*, subfamily *Alphaherpesvirinae*, genus *Varicellovirus*. PRV is the causative agent of Aujeszky’s disease (AD, pseudorabies). Although the natural host of PRV is swine, it also threatens a wide range of other mammals [10,11,12,13]. PRV has a large linear double-stranded DNA genome that encodes more than 70 functional proteins [14]. PRV Bartha-K61 strain is a classic PRV vaccine strain commonly used worldwide. It naturally lacks the gE/gI gene, and its safety and effectiveness have been widely recognized. Recently, multiple PRV-encoded viral proteins were reported to inhibit host innate antiviral response which facilitate replication and latent viral infection [15]. For example, it has been reported that the viral glycoprotein gE/gI complex reduces the phosphorylation of ERK1/2 to suppress production of type I IFNs in plasmacytoid dendritic cells [16]. PRV UL50 suppresses type I IFN signaling by promoting lysosomal degradation of IFNAR1 [17]. In addition, PRV US3 inhibits IFN signaling by promoting degradation of the host protein Bclaf1 and IRF3 [18,19]. However, research about host proteins participating in mediating PRV immune evasion is limited. 

Bcl2-associated athanogene (BAG) 3, which is a chaperone-mediated selective autophagy protein, plays a pivotal role in modulating the life cycle of a wide variety of viruses. During PRV infection, researchers found that PRV protein UL56 served as a novel BAG3 interactor by co-immunoprecipitation and co-localization analyses. The overexpression of pUL56 induced a significant degradation of BAG3 at protein level via the lysosome pathway. Overexpression of BAG3 significantly suppressed PRV proliferation, while knockdown of BAG3 resulted in increased viral replication in HEK293T cells [20]. Peroxiredoxin 1 (PRDX1) is a cellular antioxidant enzyme that is crucial for diverse fundamental biological processes, such as autophagy, inflammation, and carcinogenesis. Lin et al. reported that PRDX1 positively regulates interferon (IFN) induction and that pseudorabies virus (PRV) targets PRDX1 to evade IFN induction [21]. In addition, there are studies on the role of host restriction factors in the process of PRV infection, such as IFITM2 [22], ISG15 [23], ISG20 [24], and p53 [25]. They all play an inhibition role during PRV infection. 

Heat-shock proteins (HSPs) were a conserved protein family whose major roles seemed to promote the correct folding and assembly of target proteins as well as prevent their aggregation [26]. Heat shock protein 27 (HSP27) is a member of a small heat shock protein family; it acts as both a protein chaperone and an antioxidant, involved in the inhibition of apoptosis and actin cytoskeletal remodeling [27]. Researchers have reported that HSP27 is a ubiquitin-binding protein, mediating IκBα proteasomal degradation [28]. It also has been reported to interact with viral proteins and to be involved in viral replication [29,30,31]. Here, we demonstrate that HSP27 positively regulates PRV replication through attenuating the cGAS-STING signaling pathway.

## 2. Materials and Methods

### 2.1. Cells and Virus Strain

PK15, BHK-21, and HEK293 cells were cultured at 37 °C under 5% CO_2_ incubator in Dulbecco’s modified Eagle’s medium (DMEM) containing 10% (*v*/*v*) new bovine serum (NBS). HEK293T cells were cultured at 37 °C under 5% CO_2_ incubator in DMEM containing 10% (*v*/*v*) fetal bovine serum (FBS). PRV gE/gI-deleted vaccine strain Bartha-K61 was propagated and titrated in BHK-21 cells.

### 2.2. Plasmids and Reagents

A plasmid encoding Myc-tagged HSP27 was constructed by standard molecular cloning techniques. The expression plasmids HA-cGAS, Myc-STING, FLAG-TBK1, FLAG-IRF3/5D (the active mutant of IRF3), and Myc-Ub were all constructed in the lab. All recombinant plasmids were identified by sequencing.

The antibodies and chemical reagents used in this study were as follows: anti-Myc tag (60003-2-Ig), anti-Flag tag (20543-1-AP), anti-HA tag (51064-2-AP), anti-IRF3 (11312-1-AP), anti-HSP27 (18284-1-AP), and anti-GAPDH (60004-1-Ig) were purchased from Proteintech (Wuhan, China); anti-TBK1 (38066) and anti-STING (D2P2F) were purchased from Cell Signaling Technology (Boston, MA, USA); anti-phospho-IRF3 (AP0623) was purchased from ABclonal Technology (Wuhan, China); anti-cGAS (D163570) and anti-Ub (D220023) were purchased from Sangon Biotech (Shanghai, China); horseradish peroxidase (HRP) affinity-purified goat anti-mouse IgG (115-035-003) and anti-rabbit IgG (111-035-003) were purchased from Jackson ImmunoResearch Laboratories (West Grove, PA, USA). Lipofectamine 2000 (11668019) and lipofectamine 3000 (L3000015) were purchased from Invitrogen (Waltham, MA, USA). Evo M-MLV Mix Kit with gDNA Clean for qPCR (AG11728) was purchased from Accurate Biology (Changsha, China). TransStart Top Green qPCR SuperMix (+Dye II) was purchased from Transgen Biotech (Beijing, China). NP-40 lysis buffer (P0013F), RIPA lysis buffer (P0013K), protease and phosphatase inhibitor (P1050), Protein G Agarose (P2009), caspase 3 inhibitor Ac-DEVD-CHO (C1206) and proteasome inhibitor MG132 (S1748) were purchased from Beyotime Biotechnology (Shanghai, China). Endosomal acidification and autophagy inhibitor chloroquine (tlrl-chq) and poly (dA:dT) (tlrl-patn) were obtained from InvivoGen (San Diego, CA, USA). Western lightning plus chemiluminescence reagent (NEL105001EA) was purchased from PerkinElmer (Waltham, MA, USA).

### 2.3. Transfection and RNA Interference

BHK-21, HEK293, or HEK293T cells in 6-well plates were transiently transfected with indicated plasmids or siRNA oligos using Lipofectamine 2000 (Invitrogen, Waltham, MA, USA) according to the manufacturer’s instructions. PK15 cells in 6-well plates were transfected with indicated plasmids, siRNA oligos, or poly (dA:dT) (InvivoGen, San Diego, CA, USA) using Lipofectamine 3000 (Invitrogen, Waltham, MA, USA) according to the manufacturer’s instructions. The siRNA targeting HSP27 (siHSP27, 5′-GCUGCAAAAUCCGAUGAGA-3′) and siRNA of negative control (siNC, 5′-GUUCUCCGAACGUGTCACGU-3′) were synthesized by Guangzhou Ribo Biotechnology (Guangzhou, China). At 24 h post-transfection, the effects of HSP27 overexpression or interference were identified by Western blotting.

### 2.4. RNA Extraction and Quantitative Real-Time PCR

Cellular RNA was extracted from RNAiso Plus (Takara, Beijing, China) and transcribed into cDNA by Evo M-MLV Mix Kit with gDNA Clean for qPCR (Accurate, Changsha, China). A TransStart Top Green qPCR SuperMix (+Dye II) (Transgen, Beijing, China) was used for relative real-time quantitative PCR (RT-qPCR). The mRNA expression levels of IFN-β and cGAS were normalized to that of β-actin using the ΔΔCt method, respectively. The primer sequences used were as follows: Homo sapiens IFN-β-qF, 5′-TTGTTGAGAACCTCCTGGCT-3′; Homo sapiens IFN-β-qR, 5′-TGACTATGGTCCAGGCACAG-3′; Homo sapiens cGAS-qF, 5′-AGGAAGCAACTACGACTAAAGCC-3′; Homo sapiens cGAS-qR, 5′-CGATGTGAGAGAAGGATAGCCG-3′; Homo sapiens β-actin-qF, 5′-TGGCACCCAGCACAATGAA-3′; Homo sapiens β-actin-qR, 5′-CTAAGTCATAGTCCGCCTAGAAGCA-3′; Sus scrofa IFN-β-qF, 5′-TCCACCACAGCTCTTTCCAT-3′; Sus scrofa IFN-β-qR, 5′-CTGGAATTGTGGTGGTTGCA-3′; Sus scrofa cGAS-qF, 5′-GCACCGGGAGCTACTATGAG-3′; Sus scrofa cGAS-qR, 5′-CTCTCCACAGTGACACCTTCT-3′; Sus scrofa β-actin-qF, 5′-CAAGGACCTCTACGCCAACAC-3′; Sus scrofa β-actin-qR, 5′-TGGAGGCGCGATGATCTT-3′.

### 2.5. Co-Immunoprecipitation and Western Blotting

Cells were lysed with NP-40 lysis buffer (50 mM Tris [pH 7.4], 150 mM NaCl, 1% NP-40, EDTA, leupeptin), or RIPA lysis buffer (50 mM Tris [pH 7.4], 150 mM NaCl, 1% Triton X-100, 1% sodium deoxycholate, 0.1% SDS) containing a protease and phosphatase inhibitor (52.5 mM AEBSF, 40 μM Aprotinin, 2.5 mM Bestatin, 0.75 mM E64, 1 mM Leupeptin, 0.75 mM Pepstatin A, 250 mM sodium fluoride, 50 mM sodium pyrophosphate, 50 mM β-glycerophosphate, 50 mM sodium orthovanadate) for 30 min on ice. For immunoprecipitation assay, the supernatant after centrifugation was incubated with anti-Myc antibody or anti-HA antibody (Proteintech, Wuhan, China) for 12 h at 4 °C, and the resuspended Protein G Agrose (Beyotime, Shanghai, China) was added and incubated for 4 h at 4 °C. The complexes were centrifuged and washed 5 times with pre-chilled lysis buffer. Precipitates or cell extracts were suspended in 1x SDS loading buffer, boiled at 95 °C for 10 min, and used for Western blotting analysis. These samples were isolated in 10% SDS-PAGE gel and transferred to PVDF membranes (Merck, Darmstadt, Germany). PVDF membranes were sequentially incubated with the indicated primary antibodies and HRP-labeled goat anti-mouse IgG or goat anti-rabbit IgG (Jackson ImmunoResearch, West Grove, PA, USA). These bound protein bands were detected using Western Lightning Plus chemiluminescence reagent (PerkinElmer, Waltham, MA, USA).

### 2.6. PRV Infection and Infectivity Assays

For in vitro virus infection, treated or untreated PK15 cells or HEK293 cells were washed 3 times with PBS and infected with PRV Bartha-K61 strain (MOI = 0.001) for 24 h or 48 h in a 37 °C, 5% CO_2_ incubator. After freeze-thawing of the cell suspension 3 times, the supernatant was collected by centrifugation. The DNA was extracted from the viral stock using TIANamp virus DNA/RNA Kit (TIANGEN, Beijing, China), and the copy number of PRV UL37 gene was detected by absolute RT-qPCR using the Premix Ex Taq Probe qPCR (Takara, Beijing, China); the viral stock was serially diluted 10 times, and the PRV titers were detected in BHK-21 cells by the Reed-Muench method. The primers and probe sequences of absolute RT-qPCR used were as follows: PRV-UL37-qF, 5′-GGACTACATGTTCCCCACGG-3′; PRV-UL37-qR, 5′-TAGAACGGCGTCAGGAATCG-3′; PRV probe, 5′-(FAM) CCACGGCCGTCACGA (Eclipse)-3′.

### 2.7. Inhibitor Treatment Assay

HEK293 cells cultured in 6-well plates were transfected with Myc-HSP27 plasmid or pCMV-Myc plasmid (empty vector, EV) using Lipofectamine 2000. At 24 h post-transfection, the cells were treated with proteasome inhibitor MG132 (Beyotime, Shanghai, China), endosomal acidification and autophagy inhibitor chloroquine (InvivoGen, San Diego, CA, USA), caspase 3 inhibitor Ac-DEVD-CHO (Beyotime, Shanghai, China), or DMSO for 12 h, respectively. The supernatant after cell lysis was collected and subjected to Western blotting analysis.

### 2.8. cGAS Polyubiquitination Assay

The experiment was performed as described previously [32,33]. HEK293 cells were cotransfected with HA-cGAS, Myc-Ub, and EV or Myc-HSP27 plasmid using the Lipofectamine 2000. At 24 h post-transfection, cells were lysed and cGAS-ubiquitin complexes were immunoprecipitated with anti-HA antibody, and the ubiquitinated protein was detected with anti-Myc antibody.

### 2.9. Statistical Analysis

Data analyses were performed using GraphPad Prism 9.0 software. One-way ANOVA or two-way ANOVA were used to evaluate statistical significance. Among them, a *p* value of <0.05 was considered statistically significant, and *p* values of <0.01 or 0.001 were considered highly statistically significant.

## 3. Results

### 3.1. Overexpression of HSP27 Facilitates PRV Infection In Vitro

**Figure 1:**
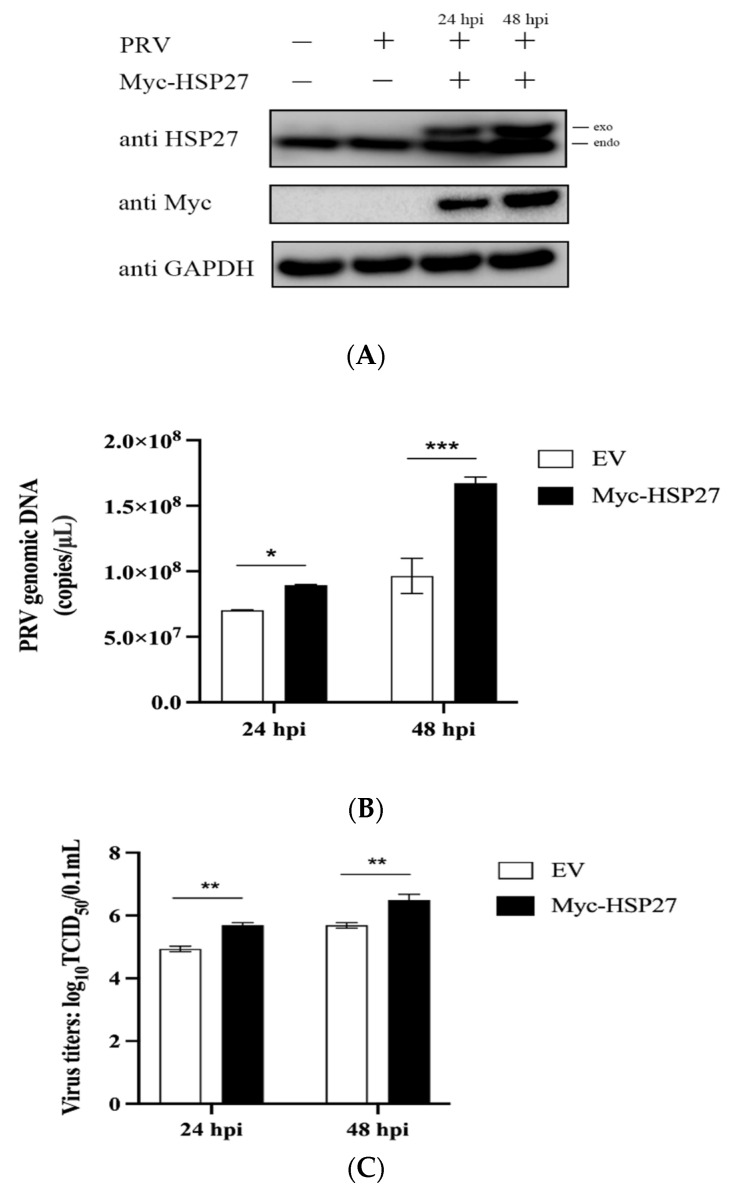
Overexpression of HSP27 facilitates PRV infection in PK15 cells. PK15 cells were transiently transfected with 1 μg of EV or Myc-HSP27 plasmid using Lipofectamine 3000 for 24 h followed by PRV infection (MOI = 0.001) for 24 h or 48 h. (**A**) Western blotting was used to detect endogenous and exogenous HSP27 proteins expression. GAPDH was used as a loading control. (**B**) Absolute RT-qPCR and (**C**) TCID_50_ assay (Reed-Muench method) were used to detect viral copy number and titers of PRV. All the data were represented as mean ± standard deviation (SD) of three independent experiments and analyzed by two-way ANOVA in B and C, * *p* < 0.05, ** *p* < 0.01, *** *p* < 0.001.

To explore the role of HSP27 in the process of PRV infection, PK15 cells were transfected with EV or Myc-HSP27 plasmid and then infected with PRV (MOI = 0.001) for 24 h or 48 h. We observed that endogenous and exogenous HSP27 proteins could be detected simultaneously only in the Myc-HSP27 transfection group, and the exogenous HSP27 protein expression showed an increasing trend with the prolongation of PRV infection time (Figure 1A). Overexpression of HSP27 significantly increased the viral copy number and titers of PRV in a time-dependent manner (Figure 1B,C), indicating that overexpression of HSP27 can promote PRV proliferation in PK15 cells.

### 3.2. Interfering with HSP27 Restrains PRV Infection In Vitro

**Figure 2:**
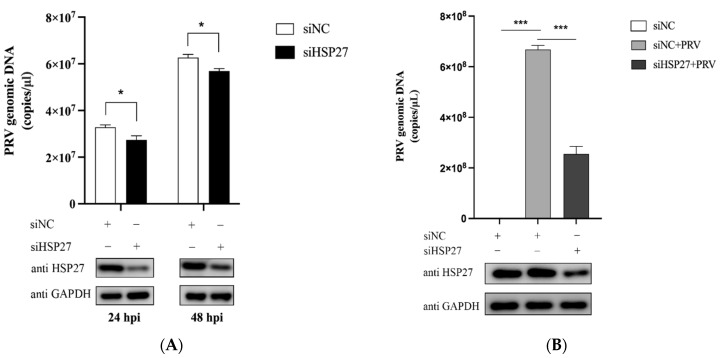
Interference with HSP27 inhibits PRV proliferation in PK15 and HEK293 cells. (**A**) PK15 cells were transiently transfected with siNC or siHSP27 using Lipofectamine 3000 for 24 h followed by PRV infection (MOI = 0.001) for 24 h or 48 h. Absolute RT-qPCR (upper panel) was used to detect the viral copy number of PRV. Western blotting (lower panel) was used to detect endogenous HSP27 protein expression. GAPDH was used as a loading control. (**B**) HEK293 cells were transiently transfected with siNC or siHSP27 using Lipofectamine 2000 for 24 h followed by PRV infection (MOI = 0.001) for 60 h. Absolute RT-qPCR (upper panel) was used to detect viral copy number of PRV. Western blotting (lower panel) was used to detect endogenous HSP27 protein expression. GAPDH was used as a loading control. All the data were represented as mean ± SD of three independent experiments and analyzed by two-way ANOVA in A or one-way ANOVA in B, * *p* < 0.05, *** *p* < 0.001.

We next transfected PK15 with siNC or siHSP27 for 24 h and then infected it with PRV (MOI = 0.001) for 24 h or 48 h. In contrast to the above results of overexpressing of HSP27, knockdown of endogenous HSP27 expression in PK15 cells reduced the viral copy number of PRV (Figure 2A), and similar results were also detected in HEK293 cells (Figure 2B). Taken together, these results suggested that HSP27 is a positive regulator during infection.

### 3.3. HSP27 Inhibits PRV Infection or Poly(dA:dT)-Activated IFN-β Expression

**Figure 3:**
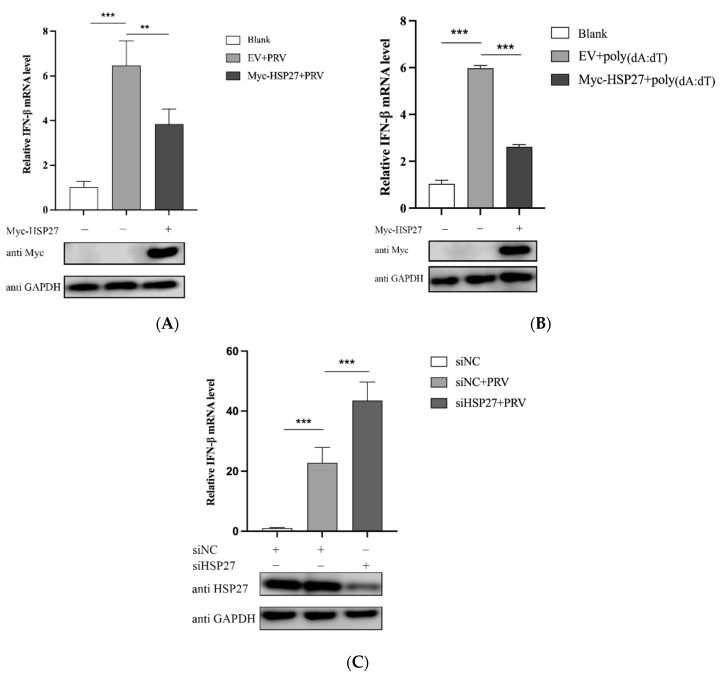
HSP27 negatively regulates PRV-triggered or poly(dA:dT)-activated IFN-β expression. (**A**) PK15 cells were transiently transfected with 1 μg of EV or Myc-HSP27 plasmid using Lipofectamine 3000 for 24 h followed by PRV infection (MOI = 0.001) for 24 h. RT-qPCR (upper panel) was used to detect the relative expression level of IFN-β mRNA. Western blotting (lower panel) was used to detect Myc-tagged HSP27 protein expression. GAPDH was used as a loading control. (**B**) PK15 cells were transiently transfected with 1 μg of EV or Myc-HSP27 plasmid using Lipofectamine 3000 for 24 h followed by poly(dA:dT) transfection for 24 h. RT-qPCR (upper panel) was used to detect the relative expression level of IFN-β mRNA. Western blotting (lower panel) was used to detect Myc-tagged HSP27 protein expression. GAPDH was used as a loading control. (**C**) HEK293 cells were transiently transfected with siNC or siHSP27 using Lipofectamine 2000 for 24 h followed by PRV infection (MOI = 0.001) for 24 h. RT-qPCR (upper panel) was used to detect the relative expression level of IFN-β mRNA. Western blotting (lower panel) was used to detect endogenous HSP27 protein expression. GAPDH was used as a loading control. All the data were represented as mean ± SD of three independent experiments and analyzed by one-way ANOVA in A–C, ** *p* < 0.01, *** *p* < 0.001.

To further explore whether HSP27 assisting PRV infection is related to IFN-β production, PK15 cells were transfected with Myc-HSP27 plasmid for 24 h and then infected with PRV. Results showed that PRV infection could indeed significantly enhance IFN-β mRNA expression level in PK15 cells; however, this activation was significantly reduced when HSP27 was overexpressed (Figure 3A). Poly(dA:dT) is a repetitive synthetic double-stranded DNA sequence of poly(dA-dT):poly(dT-dA) and a synthetic analog of B-DNA, which acts as an agonist of cytosolic DNA sensors in this study, such as cGAS [34]. PK15 cells were transfected with Myc-HSP27 plasmid before poly(dA:dT) transfection to determine the effect of HSP27 on poly(dA:dT)-activated IFN-β expression. Interestingly, poly(dA:dT) stimulated a much lower level of IFN-β expression in HSP27-overexpressing PK15 cells (Figure 3B). Moreover, we also found that the IFN-β expression activated by PRV infection was even more intense when endogenous HSP27 expression was downregulated in HEK293 cells (Figure 3C). Collectively, these results demonstrated that HSP27 negatively regulates PRV-triggered or poly(dA:dT)-activated IFN-β expression.

### 3.4. HSP27 Attenuates PRV-Triggered cGAS-Mediated Signaling Cascade

**Figure 4:**
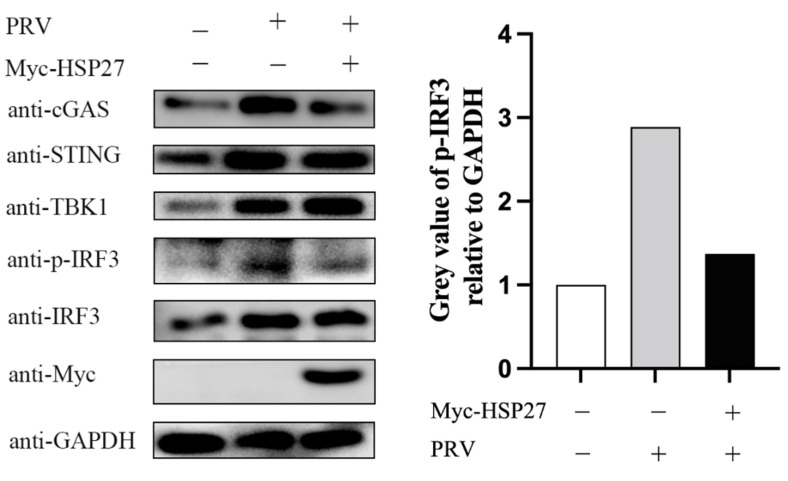
HSP27 inhibits the protein expression levels of adaptor molecules in the cGAS-STING signaling pathway induced by PRV. PK15 cells were transiently transfected with 1 μg of EV or Myc-HSP27 plasmid using Lipofectamine 3000 for 24 h followed by PRV infection (MOI = 0.001) for 24 h. Western blotting was used to detect the protein expression of cGAS, STING, TBK1, IRF3, phosphorylated IRF3, and Myc-tagged HSP27. GAPDH was used as a loading control (**left**). Greyscale analysis was used to measure the protein expression level of phosphorylated IRF3 relative to GAPDH (**right**).

Given that PRV is a DNA virus that activates IFN-β production mainly through the cGAS-STING signaling cascade [19,35], we next examined whether HSP27 affects the expression of adaptor molecules in the PRV-triggered cGAS-STING signaling pathway. As shown in Figure 4, PRV infection did enhance the protein expression of adaptor molecules in the cGAS-STING signaling pathway, such as cGAS, STING, TBK1, and phosphorylated IRF3. Furthermore, we also found that the protein expression of cGAS and phosphorylated IRF3 were dramatically reduced in the HSP27-overexpressing PK15 cells, while the protein expression of STING, TBK1, and IRF3 were not significantly different from those in the EV group. These data all indicated that HSP27 could impair the PRV-induced activation of the cGAS-STING signaling pathway.

### 3.5. HSP27 Inhibits cGAS-STING-Mediated IFN-β Expression through Targeting cGAS

**Figure 5:**
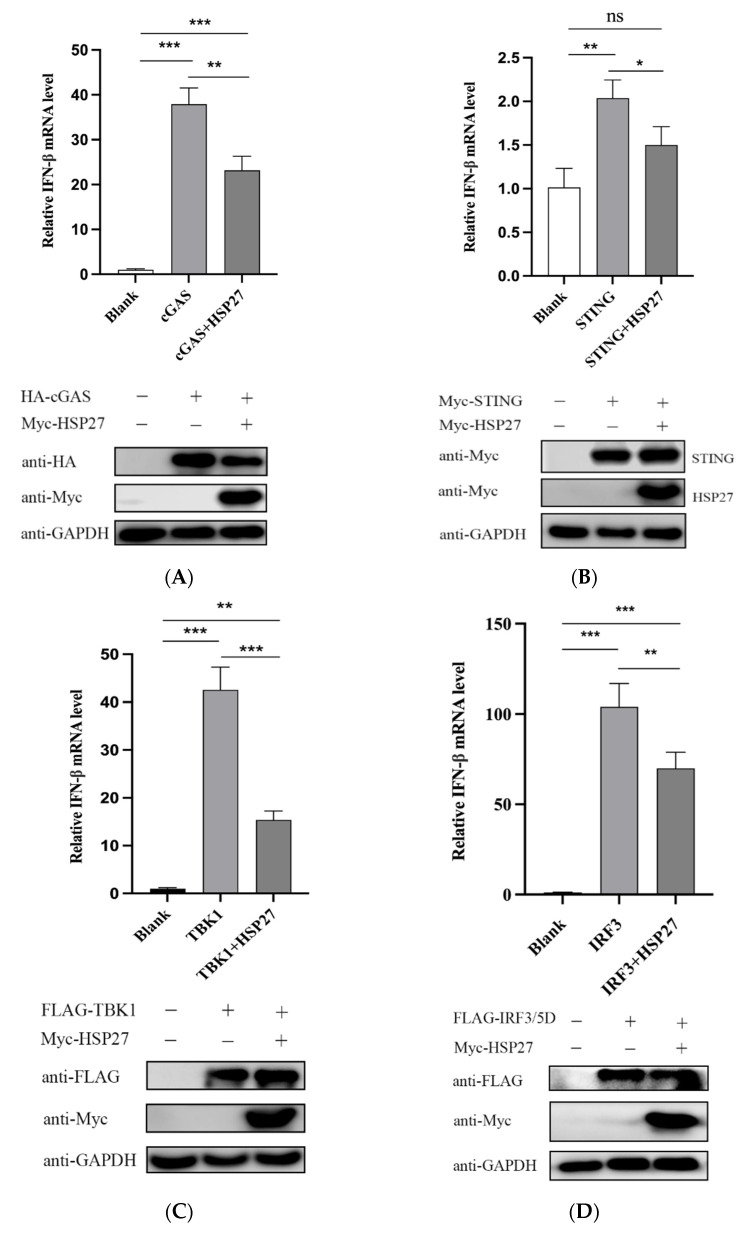

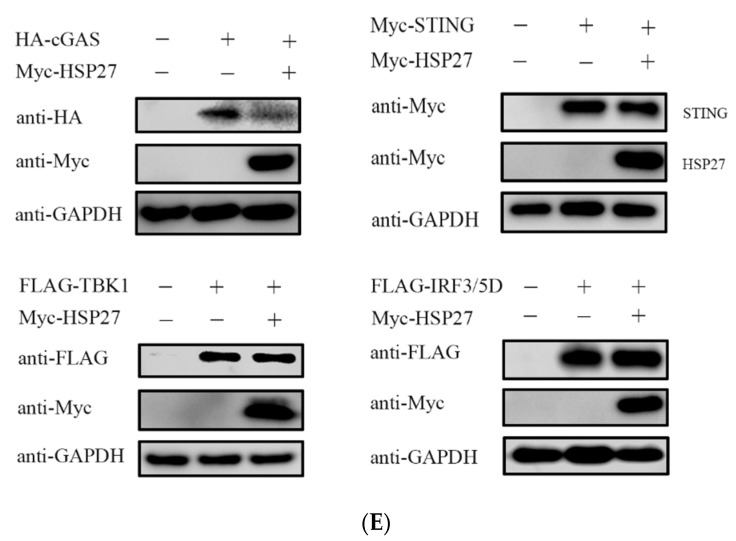
HSP27 inhibits cGAS-STING-mediated IFN-β expression through targeting cGAS. PK15 cells were cotransfected with EV or Myc-HSP27 plasmid and HA-cGAS, Myc-STING, FLAG-TBK1, or FLAG-IRF3/5D plasmid using Lipofectamine 3000 for 24 h, respectively. (**A**) RT-qPCR (upper panel) was used to detect the relative expression level of IFN-β mRNA. Western blotting (lower panel) was used to detect the protein expression of HA-tagged cGAS and Myc-tagged HSP27. GAPDH was used as a loading control. (**B**) RT-qPCR (upper panel) was used to detect the relative expression level of IFN-β mRNA. Western blotting (lower panel) was used to detect the protein expression of Myc-tagged STING and Myc-tagged HSP27. GAPDH was used as a loading control. (**C**) RT-qPCR (upper panel) was used to detect the relative expression level of IFN-β mRNA. Western blotting (lower panel) was used to detect the protein expression of FLAG-tagged TBK1 and Myc-tagged HSP27. GAPDH was used as a loading control. (**D**) RT-qPCR (upper panel) was used to detect the relative expression level of IFN-β mRNA. Western blotting (lower panel) was used to detect the protein expression of FLAG-tagged IRF3 and Myc-tagged HSP27. GAPDH was used as a loading control. (**E**) HEK293 cells were cotransfected with EV or Myc-HSP27 plasmid and HA-cGAS, Myc-STING, FLAG-TBK1, or FLAG-IRF3/5D plasmid using Lipofectamine 2000 for 24 h, respectively. Western blotting was used to detect the protein expression of HA-tagged cGAS, Myc-tagged STING, FLAG-tagged TBK1, FLAG-tagged IRF3 and Myc-tagged HSP27, respectively. GAPDH was used as a loading control. All the data were represented as mean ± SD of three independent experiments and analyzed by one-way ANOVA in A–D, * *p* < 0.05, ** *p* < 0.01, *** *p* < 0.001.

To further clarify which one or several of adaptor molecules in the cGAS-STING pathway were specifically inhibited by HSP27, we cotransfected PK15 cells with Myc-HSP27 and HA-cGAS, Myc-STING, FLAG-TBK1, or FLAG-IRF3/5D, respectively. The results showed that HSP27 clearly reduced the gene transcriptional expression of IFN-β activated by cGAS (Figure 5A), STING (Figure 5B), TBK1 (Figure 5C), or IRF3 (Figure 5D). Moreover, HSP27 significantly weakened the protein expression of exogenous cGAS (Figure 5A), and had no effect on the protein expression of exogenous STING, TBK1, and IRF3 (Figure 5B–D). Western blotting analysis of HEK293 cells also showed similar results (Figure 5E). Based on all the current results, we speculated that cGAS is a target molecule for HSP27 to attenuate cGAS-STING-mediated IFN-β production.

### 3.6. HSP27 Suppresses Endogenous cGAS Expression in Different Cells

**Figure 6:**
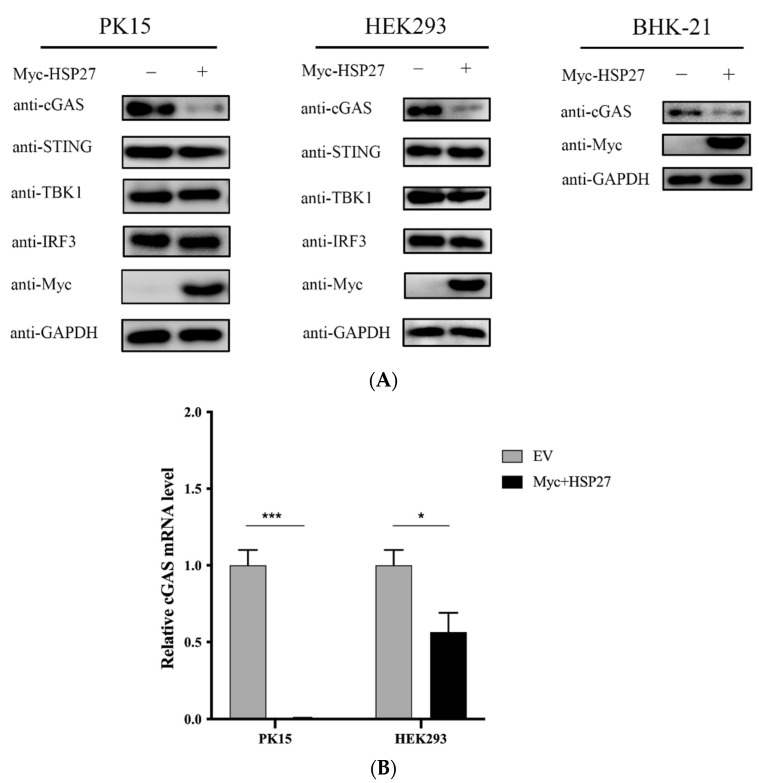
HSP27 suppresses the gene transcription and protein expression levels of endogenous cGAS in different cells. PK15, HEK293 or BHK-21 cells were transiently transfected with 1 μg EV or Myc-HSP27 plasmid using Lipofectamine 3000 or Lipofectamine 2000 for 24 h, respectively. (**A**) Western blotting was used to detect the protein expression of endogenous cGAS, STING, TBK1, IRF3, and Myc-tagged HSP27 in the above-transfected PK15 and HEK293 cells. Western blotting was used to detect the protein expression of endogenous cGAS and Myc-tagged HSP27 in the above-transfected BHK-21 cells. GAPDH was used as a loading control. (**B**) RT-qPCR was used to detect the relative expression level of cGAS mRNA in the above-transfected PK15 and HEK293 cells. All the data were represented as mean ± SD of three independent experiments and analyzed by two-way ANOVA in B, * *p* < 0.05, *** *p* < 0.001.

Next, in order to explore whether HSP27 affects the protein expression of endogenous cGAS, STING, TBK1, and IRF3, we transfected EV or Myc-HSP27 plasmid into PK15, HEK293 and BHK-21 cells for 24 h, respectively. The results showed that endogenous cGAS proteins were degraded to varying degrees in different types of HSP27-overexpressing cells, while the protein expression of other adaptor molecules did not change significantly (Figure 6A). Similar results were also detected in gene transcriptional expression of cGAS in the HSP27-overexpressing PK15 or HEK293 cells (Figure 6B). These results indicated that HSP27 represses the gene transcription and protein expression of endogenous cGAS.

### 3.7. HSP27 Interacts with cGAS and Promotes cGAS Ubiquitination

**Figure 7:**
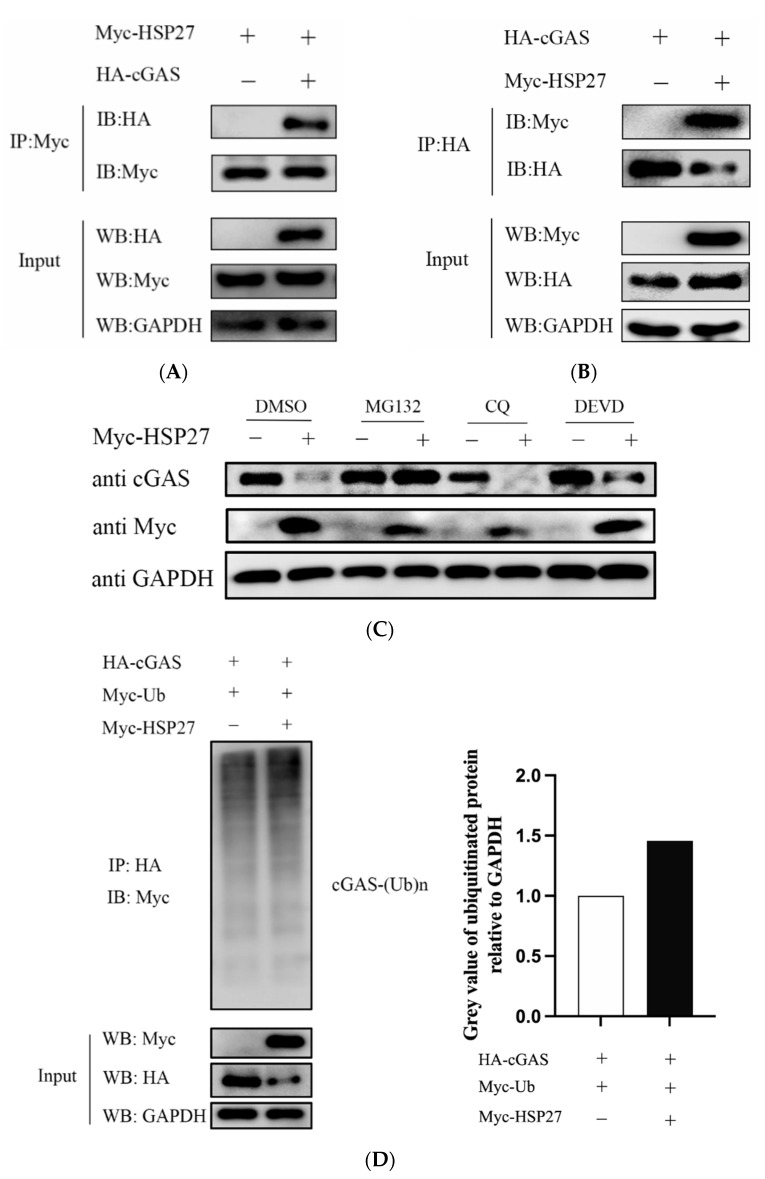
HSP27 interacts with cGAS and promotes cGAS ubiquitination. (**A**) HEK293T cells were cotransfected with Myc-HSP27 plasmid and pCMV-HA or HA-cGAS plasmid using Lipofectamine 2000 for 24 h, respectively. Cells were then lysed and immunoprecipitated with anti-Myc antibody. Western blotting was used to detect Myc-tagged HSP27 protein and HA-tagged cGAS protein in the whole cell lysates (Input) and immunoprecipitation (IP) complexes, respectively. (**B**) HEK293T cells were cotransfected with HA-cGAS plasmid and EV or Myc-HSP27 plasmid using Lipofectamine 2000 for 24 h, respectively. Cells were then lysed and immunoprecipitated with anti-HA antibody. Western blotting was used to detect HA-tagged cGAS protein and Myc-tagged HSP27 protein in the Input and IP complexes, respectively. (**C**) HEK293 cells were transiently transfected with 1 μg of EV or Myc-HSP27 plasmid using Lipofectamine 2000 for 24 h and then treated for 12 h with MG132 (7.5 µM), CQ (50 µM), DEVD (50 µM), or DMSO, respectively. Western blotting was used to detect the protein expression of endogenous cGAS and Myc-tagged HSP27. GAPDH was used as a loading control. (**D**) HEK293 cells were cotransfected with HA-cGAS + Myc-Ub + EV or HA-cGAS + Myc-Ub + Myc-HSP27 plasmid at a 1:1:1 ratio using the Lipofectamine 2000 for 24 h, respectively. Cells were then lysed, and cGAS-ubiquitin complexes were immunoprecipitated with anti-HA antibody and immunoblotted with anti-Myc antibody to detect ubiquitinated protein. GAPDH was used as a loading control (**left**). Greyscale analysis was used to measure the protein expression level of ubiquitinated protein relative to GAPDH (**right**).

To verify whether there is a physical interaction between HSP27 and cGAS, we cotransfected HEK293T cells with Myc-HSP27 and HA-cGAS for 24 h. Cells were harvested and lysed for co-immunoprecipitation experiment. The results showed that HA-tagged cGAS protein could be immunoprecipitated after coating Protein G Agarose with Myc-tagged antibody (Figure 7A). Likewise, Myc-tagged HSP27 protein could be immunoprecipitated after coating Protein G Agarose with HA-tagged antibody (Figure 7B). These data fully supported that HSP27 binds directly to cGAS.

For the sake of exploring whether HSP27-mediated cGAS degradation is dependent on the endosomal acidification and autophagy pathway, the caspase-3-dependent apoptosis pathway, or the ubiquitin-proteasome pathway, HEK293 cells were first transfected with Myc-HSP27 plasmid for 24 h, followed by treatment with endosomal acidification and autophagy inhibitor chloroquine (CQ), caspase 3 inhibitor Ac-DEVD-CHO (DEVD), ubiquitin-proteasome inhibitor MG132, or DMSO for 12 h, respectively. It was found that HSP27-mediated cGAS degradation was blocked by MG132, but not CQ or DEVD (Figure 7C), suggesting that HSP27 degrades cGAS mainly through the ubiquitin-proteasome pathway. It has been reported that ubiquitination modification can strictly regulate the stability and activity of cGAS [36], so we hypothesized that HSP27 affects the ubiquitination of cGAS, and subsequent ubiquitination assay confirmed this hypothesis (Figure 7D).

## 4. Discussion

Although the natural host of PRV is swine, it can also infect a variety of livestock and wildlife. In recent years, some cases of pseudorabies have been reported at home and abroad, but there is no conclusive evidence. In 2019, domestic researchers successfully isolated a human PRV strain hSD-1/2019 from the cerebrospinal fluid of patients for the first time [37], which provides direct evidence for the cross-species transmission of PRV from pigs to humans, suggesting that PR may have the importance of zoonosis. Therefore, it is even more urgent to clarify the molecular mechanism of PRV pathogenesis. In-depth exploration of the relationship between PRV and host proteins is of great significance for elucidating the pathogenic mechanism of PRV and anti-PRV infection.

PRV has broad cell tropism and can proliferate in a variety of cell lines, such as PK15, Vero, MDBK and BHK-21 cells. Our research group also found that PRV can replicate effectively in human-derived cell lines, such as HEK293 cells. Therefore, PK15 cells, BHK-21 cells and HEK293 cells were used in this study. The results showed that HSP27 can not only degrade the protein expression of exogenous cGAS, but also weaken the gene transcription and protein expression of endogenous cGAS. Moreover, HSP27 could degrade the expression of porcine-derived, murine-derived and human-derived cGAS, indicating that HSP27 has specificity and universality for the recognition and degradation of cGAS. Because HEK293T cells lack endogenous cGAS [38], HEK293T cells are mainly used for co-immunoprecipitation assay of exogenous cGAS and HSP27. Subsequent results showed that HSP27 directly binds to cGAS and promotes the ubiquitination and degradation of cGAS. There are about more than 70 kinds of structural and nonstructural proteins in PRV [14]. Although the roles of some viral proteins in viral infection have been confirmed [15,16,17,18,19,32,35], the role of a considerable number of viral proteins remain unknown. Further studies are needed to confirm whether the viral proteins of PRV participate in the regulation of HSP27 on cGAS-STING signaling pathway to achieve active immune escape.

Many studies have shown that HSP27 regulates the proliferation of RNA viruses, mainly through the NF-κB signaling pathway, RLR signaling pathway, or autophagy pathway. HSP27 promotes the replication of foot-and-mouth disease virus (FMDV) by interacting with the structural protein VP2 of FMDV and activating the autophagy-related pathway EIF2S1-ATF4-AKT-MTOR [39]. HSP27 inhibits the proliferation of encephalomyocarditis virus (EMCV) by stabilizing the expression of MDA5 and thus positively regulating the RLR/MDA5 signaling pathway [31]. HSP27 negatively regulates the replication of classical swine fever virus (CSFV) by directly interacting with non-structural protein NS5A and promoting the activation of the NF-κB signaling pathway [40]. However, there are few studies on the mechanism of HSP27 regulating DNA virus proliferation. This study is the first to report the relationship between HSP27 and cGAS, a cytosolic DNA sensor, which provides a reference for future research of HSP27 in DNA viruses. Given that HSP27 is a multifunctional protein that can participate in multiple immunomodulatory processes of the body, whether it also regulates other signal pathway related molecules in PRV infection still needs to be further explored. HSP27 contributes to PRV infection, so HSP27 inhibitors are expected to be candidates for PRV-targeted therapy, and HSP27 stable cell lines may also be used in large-scale culture and proliferation of PRV.

There are four main modifications of cGAS: ubiquitination, phosphorylation, acetylation, and palmitoylation [36,41,42,43]. Among them, ubiquitination modification is an important form of protein post-translational modification, which can regulate and change the properties and functions of proteins, such as activity and stability. It is widely involved in many physiological processes, such as protein degradation, cell cycle regulation, immune response regulation, and DNA damage repair. Here, we report that HSP27 attenuates the cGAS-mediated interferon signaling cascade by promoting cGAS ubiquitination and proteasomal-dependent degradation. K48-linked ubiquitination modification is the most common of all ubiquitin chains, accounting for about 50% of all ubiquitination modifications, and it is closely related to proteasomal degradation. Therefore, it is speculated that the way that HSP27 ubiquitinates cGAS may be K48-linked, which requires further investigation.

**Figure 8:**
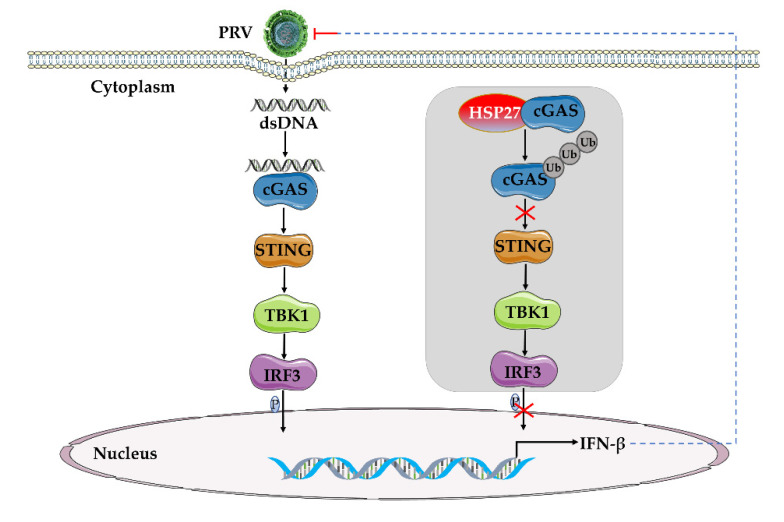
Schematic model of HSP27 negatively regulating cGAS-STING-mediated IFN-β expression upon PRV proliferation. cGAS recognizes the dsDNA of PRV and then activates cGAS-STING-mediated IFN-β signaling pathway. HSP27 directly binds to cGAS, promotes cGAS degradation through ubiquitination, attenuates cGAS-STING axis signaling transduction, and inhibits IRF3 phosphorylation and IFN-β expression, thereby promoting PRV infection.

In this study, we found that overexpression of HSP27 contributed to PRV infection, and knockdown of HSP27 suppressed PRV infection in vitro. HSP27 inhibited PRV infection or poly(dA:dT)-activated IFN-β expression. Interestingly, we found that HSP27 attenuated cGAS-STING-mediated IFN-β expression through targeting cGAS. Further studies found that HSP27 bound directly to cGAS and promoted cGAS degradation by ubiquitination, and impaired the cGAS-mediated signaling cascade and IFN-β expression, thereby promoting PRV infection (Figure 8). In conclusion, we demonstrate for the first time that HSP27 is a novel negative regulator of the cGAS-STING signaling pathway, reveal a novel mechanism by which HSP27 facilitates PRV infection, and confirm that HSP27 plays a crucial role in PRV infection.

## Data Availability

All available data are presented in the article.
